# RE-AIMing conferences: evaluating the adoption, implementation and maintenance of the Rick Hansen Institute’s Praxis 2016

**DOI:** 10.1186/s12961-019-0434-1

**Published:** 2019-04-11

**Authors:** Heather L. Gainforth, Kristy Baxter, Justine Baron, Emilie Michalovic, Jeffrey G. Caron, Shane N. Sweet

**Affiliations:** 1 School of Health and Exercise Sciences, University of British Columbia Okanagan, Kelowna, BC Canada; 2 International Collaboration on Repair Discoveries (ICORD) University of British Columbia, Vancouver, BC Canada; 3 Ottawa Hospital Research Institute, Ottawa, ON Canada; 4 Department of Kinesiology and Physical Education, McGill University, Montreal, QC Canada; 5 Center for Interdisciplinary Research in Rehabilitation of Greater Montreal (CRIR), CanadaCenter for Interdisciplinary Research in Rehabilitation of Greater Montreal (CRIR), Montreal, Quebec Canada; 6 School of Kinesiology and Physical Activity Sciences, Université de Montréal, Montreal, QC Canada

**Keywords:** Praxis, Knowledge translation, RE-AIM framework, Evaluation, Spinal cord injury

## Abstract

**Background:**

In April 2016, the Rick Hansen Institute (RHI) hosted an innovative, 2-day conference called Praxis 2016. RHI aimed to bring together a diverse group of stakeholders to develop solutions for overcoming the challenges of translating spinal cord injury (SCI) research into practice. To understand the impact of Praxis, RHI funded an independent team to evaluate Praxis at the individual and setting level using the RE-AIM framework. Individual-level findings are published elsewhere. The aim of this evaluation is to report on the impact of Praxis at the setting level in terms of its adoption, implementation and maintenance.

**Methods:**

Data sources included interviews with attendees (*n* = 13) and organisers (*n* = 9), a fidelity assessment conducted at the conference, and observation notes provided by the evaluation team.

**Results:**

Main findings indicated that the Praxis model was adopted by organisers and attendees, implemented by RHI as intended, and has the potential for long-term maintenance. Lessons learned highlighted the importance of including SCI community members throughout the entire process from development to post-conference activities as well as in the research process, the value of facilitation and fostering interactive problem solving, and the need to identify leadership and funds to foster long-term efforts.

**Conclusions:**

Developing and implementing a solutions-focused conference that brings together a diverse group of SCI stakeholders was challenging and rewarding for attendees and organisers. Other domains could learn from, adopt and build on the Praxis 2016 approach to address research-to-practice gaps.

**Electronic supplementary material:**

The online version of this article (10.1186/s12961-019-0434-1) contains supplementary material, which is available to authorized users.

## Background

It is estimated that traumatic spinal cord injury (SCI) costs the Canadian government approximately $2.7 billion per year, with an estimated average lifetime cost per individual of $1.5 million for incomplete paraplegia to $3.0 million for complete tetraplegia [1, 2]. While funding is provided to conduct research to enhance the lives of people with SCI, few research findings are translated into practice [3]. Indeed, the 17- to 20-year gap between bench-to-bedside and bedside-to-real world application is so difficult to overcome, that they are often referred to as the “*valleys of death*” [3, 4].

The Rick Hansen Institute (RHI) is a Canadian-based, not-for-profit organisation that is committed to accelerating knowledge translation (KT) of discoveries and best practices into improved outcomes for people with SCI [3]. In April 2016, RHI developed and hosted an innovative conference called Praxis 2016 (https://rickhanseninstitute.org/work/praxis/model/88-rhi-praxis-model-no-comment-allowed/448-praxis-2016-conference). Praxis had three primary goals, namely to (1) develop a shared understanding and synergy concerning KT and desired outcomes; (2) foster the sharing of real-world experience between attendees, and (3) identify actions to overcome the barriers and challenges to achieve KT. In terms of format, RHI aimed to change the mould of traditional scientific meetings. To foster interdisciplinary thinking, RHI invited a diverse group of adults living with SCI, researchers, clinicians, funding organisations, regulators, policy-makers and industry partners to attend the conference. Rather than following a didactic format, the conference included presentations on speakers’ translational lessons learned, interactive panel discussions, and working table discussions [5]. All conference topics were first presented by three to four expert speakers per topic area. Rather than following a traditional conference style, speakers were asked to outline their translational lessons learned. Speakers were then asked to engage in a panel discussion about their lessons learned, which was moderated by the conference facilitators. Finally, a working table session was held in which attendees worked in assigned groups of 10 to develop solutions to the barriers outlined by the speakers. To guide future KT efforts in SCI research, strategies, opportunities, tactics and recommendations developed during Praxis were to be synthesised into a conference report [6] and action plan [7].

To learn from, improve and inform future KT initiatives, RHI funded an independent evaluation team to examine the impact of Praxis. The evaluation was guided by the RE-AIM evaluation framework, which captures five factors associated with measuring impact – Reach, Effectiveness, Adoption, Implementation and Maintenance [8]. Reach and effectiveness were conceptualised as individual-level factors, adoption and implementation as setting-level factors, and maintenance as both an individual and setting level factor. This paper focuses on setting-level factors (i.e. adoption, implementation and maintenance). Adoption refers to the proportion and representativeness of settings and/or groups that are willing to adopt the initiative. Implementation examines the extent to which the initiative was delivered as intended. Maintenance refers to the extent to which the initiative becomes part of an organisation or groups’ long-term practices or policies [8, 9]. By examining these setting-level factors, other teams can learn about the successes and challenges of developing and implementing large-scale, event-based KT initiatives.

A separate paper reported on the individual-level impact of Praxis on attendees in terms of reach, effectiveness and maintenance. Overall, findings indicated that Praxis reached a wide range of attendees, but further representation from the SCI community was needed. Praxis improved attendees’ knowledge about KT barriers and solutions and increased the number of groups attendees believed they needed to work with to translate research. Given that setting-level factors can help to explain and translate individual-level successes and failures [9, 10], this paper reported on the impact of Praxis at the setting level (i.e. the RHI and Programme Advisory Committee (PAC)) in relation to adoption, implementation and maintenance. Specifically, we examined how speakers and organisers adopted the Praxis 2016 model, the extent to which Praxis 2016 was implemented as intended, and how Praxis could have a long-term impact in the SCI field.

## Methods

### Praxis 2016

Praxis 2016 consisted of a 3-day conference held in Vancouver, BC (April 25–27, 2016). A detailed description of the conference is provided in Additional file 1.

### Evaluation procedures

Setting-level data were obtained from five different sources, as follows: (1) semi-structured interviews with attendees; (2) semi-structured interviews with organisers, referring to RHI staff and the PAC; (3) fidelity assessment; (4) the evaluation team’s notes; and (5) observations of teamwork at working table sessions. Cost-effectiveness data were not available. Given this project is a programme evaluation, ethics approval was not required; however, informed consent was obtained from all interview participants and raw data were only accessed by members of the evaluation team. Procedures associated with data collection and data analyses for each source are outlined in Additional file 1.

### Data analysis

Findings from all five data sources were used to evaluate Praxis 2016 in terms of the RE-AIM domains of adoption, implementation and maintenance. Data sources associated with each dimension are outlined in Table 1.

**Table 1:** Data sources associated with AIM dimensions

Dimension and operational definition	Attendee interviews	Organisers interviews	Fidelity assessments	Evaluation team notes	Table observation
Adoption: rationale for organisers’ and speakers’ involvement in Praxis		✓			
Implementation: extent to which Praxis was delivered as intended as well as challenges and facilitators that influenced the process	✓		✓	✓	✓
Maintenance: perceptions of the long-term impact of Praxis on both spinal cord injury stakeholders and Rick Hansen Institute	✓	✓			

All interview guides are provided in Additional file 2. Interviews were audio-recorded, transcribed verbatim and subsequently analysed using both deductive and inductive approaches. First, one evaluator (KB) familiarised herself with the data by reading the transcription while listening to the recording. KB then deductively analysed the data into higher-order themes of adoption, implementation and maintenance (i.e. the ‘AIM’ in RE-AIM). Specifically, KB identified blocks of text from the interview transcripts that were relevant to the adoption, implementation or maintenance of Praxis 2016. Once the 22 transcripts were initially organised deductively into higher-order themes, KB then inductively analysed the data to search for lower-order themes within each of the higher-order themes. For this part of the analysis, we followed Braun and Clarke’s six-phase process for thematic analysis [11, 12]. Lower-order themes were coded through a collaborative and iterative process involving three evaluators (KB, HG, SS). Specifically, the definitions and names (labels) of the lower-order themes were discussed over the course of seven meetings (approximately 13 h). To finalise the analysis, each higher- and lower-order theme was given a description to ensure that each was distinct from the rest of the themes in the analysis.

Seven evaluators used a fidelity assessment tool based on the conference agenda to evaluate implementation. All session tasks and goals were marked as either complete or incomplete by all seven evaluators. If > 50% of the evaluators marked the task or goal as complete, it was considered completed. A percentage completion score (i.e. the percentage of tasks completed) was also calculated for each session on completion of all goals.

All evaluation comments collected from the fidelity assessment tool were transcribed verbatim in an Excel sheet. Only comments related to implementation were thematically analysed. Consistent with Braun and Clarke’s [11, 12] recommendations, one researcher (KB) first assigned codes to each comment. She then reviewed the codes to identify key themes and subsequently definitions for each theme. Finally, the researcher worked with the lead evaluators (HG and SS) to refine themes.

Working table teamwork scores were assessed using a modified version of the Jefferson Teamwork Observation Guide [1] (JTOG) (Additional file 3). JTOG scores were calculated by creating a sum of all JTOG competencies for each table (maximum score: 56). To allow for descriptive comparisons, mean, mode and ranges were calculated for the conference overall as well as for each session.

## Results

### Attendee interviews

Thirteen attendees (53% women, mean age 48 years, SD 15 years) were interviewed following the Praxis 2016 conference. A diverse group of attendees indicated they wanted to be interviewed, including two individuals with SCI, two KT specialists, three researchers, one industry partner, three members of the SCI community, one policy-maker and one clinician. All interviews were completed within 3-months post-Praxis (mean duration 40 min, SD 7 min). Key themes, associated definitions and illustrative quotes are outlined in Table 2.

**Table 2:** AIM themes and sub-themes derived from interviews with Praxis attendees

Themes and definitions	Subthemes and definitions	Exemplar quotes
**Implementation:** • At the setting level, implementation refers to the intervention agents’ fidelity to the various elements of an intervention’s protocol, including consistency of delivery as intended and the time and cost of the intervention • At the individual level, implementation refers to clients’ use of the intervention strategies		
Perceptions around the purpose of the conference: attendees’ understandings and perceptions of the purpose of the conference	Lack of clear understanding beforehand: there was a lack of clear understanding of the goals of the conference before attendees arrived	• “*… perhaps, to be improved, is getting people more focused before they arrived so that they’re really aware of what the format was going tobe*” • “*I didn’t receive any material about the content of the conference until immediately before, so I assumed that the conference was largely going to be about presentations* [and therefore] *I scheduled their work time to be present for the presentations* [and was] *absent for the working sessions. The value I could have added, I wasn’t actually at the meeting for that*”
	After attendees’ perceptions were clarified: there were two perceived goals that those who attended the conference felt Praxis was trying to achieve: (1) creating collaborative networks (2) developing solutions to overcome challenges	• “*Well, I think they were trying to achieve the collaborative conversations and engagement from all of the stakeholders*” • “*Oh, well I took Praxis to be actually trying to come up with solutions to the problems that we know exist*”
Working table sessions (interactive environment): the working table sessions both facilitated and inhibited the two conference goals	Aspects of the interactive environment related to networking: aspects of the working table sessions that either facilitated or inhibited the conference goal of networking	• “*It was the first conference I’d ever been at where we were seated at particular tables, which I think actually was a good idea because it breaks up groups that might attend conference together and it forces you to mix with other people*” • “*… I would’ve preferred the opportunity to move around instead of just staying at one table … But if the real goal was to increase the communication and understanding between stakeholders, it would have been good to shake that up more*”
	Aspects of the interactive environment related to creating solutions: aspects of the working table sessions that either facilitated or inhibited the conference goal of creating solutions	• “*But if they’re trying to get solutions, they probably would’ve gotten a lot further faster if people had been grouped with* [similar roles]*,right?*” • “*… I mean, one of the challenges was a lot of the problems are so big and systemic that in 5 min you can’t really come up with a good solution*” • “*At my table everybody was engaged … I thought the conversation was just flying really well, where people were just putting their ideas out there, nobody was really interrupting anybody either*”
**Maintenance:** • The extent to which a programme or policy becomes institutionalised or part of the routine organisational practices and policies • Within the RE-AIM framework, maintenance also applies at the individual level • At the individual level, maintenance has been defined as the long-term effects of a programme on outcomes after 6 or more months after the most recent intervention contact		
Future directions: Attendees spoke of hopes, recommendations and concerns post-Praxis; their expectations for the future impact of Praxis	Hopes for the future: people had expectations of how Praxis would change the spinal cord injury field	• “… [having] *the goal to better understand the problem and recognize that we’ll all go back to our areas of influence with a better understanding of the problem,* [that’s] *probably a bit more effective if we’re trying to bring about change. Then something like that is helpful*” • “*So, having a funder like RHI to come in and say, ‘these are all great ideas, but how are we actually going to implement them … could be for who knows what … that’s what I hope comes out of it is that there’s a focus from RHI to really drive this collaborate process that they started at Praxis*”
	Recommendations for ensuring Praxis has impact: these are proposed solutions that attendees have post-Praxis to make the action items actually happen	• “*… an email of who was doing what and not just of what was happening … should be able to offer assistance … would be a good way of including everyone from the conference*” • “*I think what the whole conference needs to do is really take all of the information from the meeting and people’s comments and develop some very short term, concrete action items*” • “*… value of creating, fostering, supporting real partnerships amongst the stakeholders that are interested in providing treatment, innovative treatment options for the paralysis community, I think that’s going to be necessary is we’re going to get there* [goals of the conference]”
	Concerns about impact: attendees were sceptical that there would be no real change over the long term	• “*… we had good discussion, we came up with some good ideas, some good plans. But we were a little frustrated, that you know, basically going to write a paper, report back and that’s going to be it*” • “*I think the conference to me highlighted a number of challenges, but I didn’t actually hear solutions to the challenges or new approaches to solving the problem*”
Continuing to bring people together: attendees 1expressed a need to continue to bring people together post-conference in different sorts of ways	Need for leadership: attendees described a need for leadership post-Praxis to make the action items happen	• “*Having a funder like RHI, or whoever, to come in and say, ‘these are all great ideas, but how are we actually going to implement them?’*” • “*… from my perspective moving forward, there was an identifiable person or group of people that the whole community could look to and say, ‘oh we just have to talk to them and they could get us to the right information’ and ‘we can talk to them and find out about this new research project’*”
	Value in getting diverse groups together long term: attendees described the value of getting diverse groups together (face-to-face) to move the SCI field forward after Praxis	• “*Communication is always gonna be the critical push. So, I hope Praxis is the beginning of a push for Rick Hansen* [Institute] *to do more of these types of groups*” • “*… people will have to get together to have a bigger force … that’s one thing that was named to have more, you know, research groups or whatever, work more together to join forces and make things happen faster*”

Headings are set in bold

Regarding implementation, attendees expressed challenges related to understanding the unconventional goals of the conference before arrival. However, goals related to creating collaborative networks and developing solutions to translational challenges became clearer at the conference. Attendees expressed mixed opinions regarding the working tables and presentations. Some attendees found the assigned seating limiting; whereas, others felt that the tables were beneficial and expanded their networks. Some attendees also felt that the speakers’ presentations should have been shorter, allowing for more time for working table activities.

Regarding setting-level maintenance, after the conference attendees felt that Praxis 2016 would help to highlight challenges and solutions for bridging the ‘valleys of death’ long term; however, attendees were sceptical as to whether solutions would be implemented without strong leadership and input by diverse groups, including members of the SCI community. Themes related to adoption were not derived.

### Organiser interviews

Five RHI staff and four members of the PAC participated in a telephone interview (66% women, mean age 52 years, SD 9.7 years). Five interviews were conducted by HG and four by SS (mean duration 52 min ± 11 min). Key themes, definitions, and illustrative quotes are outlined in Table 3.

**Table 3:** AIM themes and sub-themes derived from interviews with Rick Hansen Institute (RHI) staff and Programme Advisory Committee (PAC)

Themes and definitions	Subthemes and definitions	Exemplar quotes
**Adoption:** • How did RHI and the PAC develop organisational supports to deliver Praxis? • Information about experts or speakers choosing to adopt the Praxis programme • RHI & the PAC’s decision to adopt the Praxis model		
PAC/RHI members reasons for adopting Praxis: the goals of Praxis aligned with members’ personal and/or professional interests	N/A	• “*Considering that we really wanted to ensure that Praxis brought together the perspectives of a wide variety of stakeholders, and* [my position being useful]*, and our desire to ensure that people with spinal cord injury had a strong voice in the proceedings, it seemed only natural that I should be part of the pro-planning process*” • “[I joined] *because I thought what they were trying to do was important. And I wanted to ensure that our community, like rehabilitation community, got to play an active role in the process*”
Adopting the unique format of Praxis: there was a mixture of hesitance and willingness across a variety of individuals to adopt the unique format of Praxis	Hesitance to adopt the format: the PAC/RHI members speak of the unease some stakeholders had with the format of Praxis	• “*And a number of these individuals were quite hesitant to really be completely engaged, like okay well they’re willing to come and speak but they didn’t see themselves as being advisors to the programme and subsequent activities*” • “[The Facilitator] *opened by saying, ‘Okay. So, we’re not expecting PowerPoint. And if you do PowerPoint, we’re talking about three or four slides and we want discussions and case review. You are to bring your wisdom and experience*.*’ And there was complete silence*” • “*But with sponsors, it was really tough to explain it. We did not get sponsorship because we would not provide a standard exhibit space because we would not allow them the chance to address the delegates and do sunrise breakfast and things like that*”
	Acceptance of the format: the PAC/RHI members speak of how some stakeholders received the Praxis format	• “*What I’m so humbled by is the fact that they were willing to join in and go along with something that they couldn’t feel. They didn’t have any evidence, they didn’t have any examples and I just had so much admiration for the fact that everyone just dug in and did the planning without any idea of how this would turn out*”
**Implementation** • Did RHI and the PAC deliver Praxis as intended? • What were the challenges to implementing Praxis? • What were the facilitators in implementing Praxis (i.e. what went well)?		
Delivery: aspects of the conference discussed that are related to the ability to deliver the conference as intended		
Interactive format: organisers spoke of their satisfaction with the resulting collaborative format of the conference.	N/A	• “*And in that, sort of when you were thinking about speakers then, one of the key things was to find people that were engaging, that weren’t just necessarily experts but could also engage a large audience*” • “[Yes]*, that comes into my decision making*” • “*So, we did that* [seating charts] *for the conference, and I think that was a very valuable exercise because it forced people to sit with other people who* [they] *would not normally be sitting with*”
Snowball reach: the invitation process occurred through the use of a snowball method	N/A	• “*Organically. I say that evolved. So, we didn’t have, at the beginning, a strict criteria. We were starting from nothing. So, it became friends of friends of friends*”
The action plan was difficult to create/deliver: the vastness of the information within the action plan made it difficult to formulate and subsequently deliver	N/A	• “*I think it comes down to planning and resourcing it, really insufficient resources to get the action plan out in that time frame, considering the barriers to actually getting the information together, synthesizing – I don’t know if we thought we would get so much information from the participants either. It took a lot of work just to synthesize it*” • “*It* [the action plan] *is so diverse and so many recommendations …. there’s a lot of themes, there’s a lot of work*” • “*Just saying I think some of the aspirational goals, they’re very lofty – to me, some of the things that are in Praxis are 10-year goals, which I don’t disagree with the aspirational goals, but I would have liked to have seen some more near-term, pragmatic, what will we achieve?...*”
Challenges: difficulties the PAC/RHI members experienced during the planning of Praxis		
Iteration throughout planning: there was a lack of clarity of the goals and format at first, leading to a variety of planning challenges	N/A	• “*And then ultimately, personally I wasn’t quite sure what the goal was for the meeting … We need to know what was* [the] *goal* [is] *in order to provide the most constructive input*” • “*I think the conference that we ended up with was probably very different than what was originally envisioned when we started. And I think that’s a positive thing, I think it evolved in a very positive way*” • “*I don’t know if there was a real plan for the conference. I think it was really a progression, and I can’t even say who got us to the point where we needed to focus on solutions*”
Lack of resources: a shortage of time and staff to plan praxis	N/A	• “*I think, you know, it was internally, it was a lot of work you know, I think everybody involved put in a lot a lot of hours, they put their soul into it, and by the end they were utterly exhausted …, I think that there was a significant amount of burnout in the immediate aftermath of the conference. Just because you know people were working beyond capacity for so long in order to get the conference going*” • “*I think the process felt rushed at times, the planning process. I think once we got on that track, there was limited time to get it done … It did feel like a tight time frame considering that it wasn’t something that a lot of us had done before*”
Selection of speakers: the selection of speakers was difficult due to the uniqueness of the format	N/A	• “*The challenge of finding speakers is that they were disorganized in the beginning and so if you want the best speakers, their schedules are very tight and so you really need to have you know, a lot of time to be able to get the people that you want*” • “*I think that you know the speakers, that some of the speakers were chosen early on and perhaps were not the best fit in the end*”
Accessible conference: difficulties associated with the planning of an accessible conference	N/A	• “*So, I think my most important lessons apart from all the conference stuff about who I’d use and who I wouldn’t use is a warning that the minute you say it is accessible, you are opening a Pandora’s box. And it is going to cost more and it’s going to take more thought and more time. And I don’t only mean accessibility for people who have disabilities, I mean accessibility in allowing everyone’s voices to be heard*”
Facilitators: aspects that facilitated the planning and/or implementing of the conference		
Strong leadership: it was helpful to have strong leaders who could ensure buy in from the broader spinal chord injury (SCI) community	N/A	• “*We started to feel more comfortable … we certainly were feeling more valued in our input …* [the leader] *knows more of the big picture and … had the ability to make decisions*”
Value of facilitation: Facilitators were very important in the planning of the conference to ensure implementation was successful	N/A	• “*Definitely having the facilitators there was a really effective piece of the planning. And in the product of the actual meeting …. I think they were probably the neutral people. If a topic came up that was getting off topic or was decided ahead of time that we’re not going to be discussing this, then they stopped that immediately so it didn’t take time away from what we were trying to discuss*” • “*At the meeting itself, they were wonderful facilitators. They really were. And just watching them perform and do their thing.* [They] *were making sure that the presentations seemed to be ending with a clear message or wrapping up in a certain way. And even if it hadn’t all been planned ahead of time, they were making it happen on the spot … And they did it with very good cheer if you will*”
Initiatives to unite consumers: extra steps were taken to ensure consumers felt united before the conference	N/A	• “*I know the organizing committee made sure that there was a consumer at every small table*” • “*The initiative* [was made] *to arrange a networking meeting for all of the individuals that fell under that umbrella of consumers to get together in an immediate proceeding before the conference actually opened.* [The motive was] *to bring everybody together to get to know each other and develop networks, but* [they were also] *charged to speak up, to get involved in the conversation, to not be intimidated by any of the other attendees and to make sure that they knew that their opinions were valid and that it was important that they make themselves be heard and be part of the conversation, a part of the solution that Praxis was trying to achieve*”
Satisfaction of the organisers: the organisers spoke of feelings of satisfaction and dissatisfaction with the conference		
Exceeded expectations: organisers spoke of how the conference exceeded their initial expectations	N/A	• “*So, I thought that was good* [table discussions]*, we needed to have those discussions and I was somewhat hesitant as to whether or not you know, those discussions would lead to anything in the future*” • “*And, you know, and then as I say, the conference I think more than lived up to everyone’s expectations*” • “*In the end, I think the meeting came off as well as expected, and in some cases, better than expected*”
Disappointing long-term impact: organisers spoke of how they were disappointed with the impact of praxis long term	N/A	• “*There’s also probably a lot of people that went back to work and got caught up in their everyday challenges*” • “*I’ll just say from my very arms-length perspective I haven’t really seen anything moving forward as a result of Praxis … And, I don’t know that that has happened*”
**Maintenance** • What are the long-term impacts for attendees? The SCI Community? RHI? • How has Praxis changed RHI’s organisational practices? • What might be changed in the implementation for a future Praxis-type meeting?		
Recommendations post-Praxis: suggestions for fostering impacts of Praxis	Leadership for implementation of the action plan: there needs to be coordination of who is in charge of the action plan that results from Praxis	• “*I think that something like the Rick Hansen Institute … is the only organization that is positioned to make something like that happen* [another Praxis type meeting]*. Just because of our sort of neutral position amongst all of the key stakeholders and our key role as a network development organization. I think that if solutions were to be developed in a sort of collaborative fashion, that it would be instrumental to have RHI sort of facilitate that*” • “*You know, one of the problems with moving things forward is you need a champion to say, ‘this is something that I want to see happen’. So, really you know I think the challenge is that if you want these things to have an impact, somebody needs to want to make an impact*”
	Continuing to meet face-to-face: it is important for these face-to-face meetings to continue happen to foster the impact of praxis	• “*We’ll probably have another Praxis-like conference and we will probably have some smaller Praxis-like conferences as well … Yeah, I think that what would be perfect actually is to reconvene and talk about you know where we are, where the gaps are and you know following these initiatives - how can we make you know this action plan that we have?*” • “*But you know, you can read as much as you want and you know you can read policies or you can read grant applications which require consumer engagement. But unless you are in a room with other consumers I think the most powerful thing was having this face-to-face with consumers and listening to their points of view*”
	Prioritising and creating short- and long-term goals: there needs to be clear short- and long-term goals created and priorities must be set	• “*But I would like to think that RHI over the next 5 years would use the action plan developed from Praxis as a basis for developing its business plan and try to identify some of the challenges in the action plan and look to try to find solutions for them*” • “*I think the development of the action plan of as well or the action plan itself will be key in RHI’s business planning moving forward. It’s a list of things that the field is not doing well right now, the action plan or the field needs. And RHI’s role as an institution is to fill gaps. And if we’re not following the action plan, I think we’re failing the SCI community*” • “*I think it might be better to do smaller scale, feasible things than have people join a successful group, as opposed to waiting for a larger group to coalesce on a common thing*”
	Involving consumers: SCI consumers must be involved to foster the impacts of Praxis	• “*So, packing into the SCI community to share data, disseminate data and information and to gain input on the design of trials or the design of implementation. Actually, having them* [consumers] *as partners in all of these different aspects could really, I mean and they can enhance recruitment for other trials. They can spread the word about clinical interventions that are trying to be implemented into hospitals or clinical care. They can be influential on their communities about that. I mean there’s so many ways that people with spinal cord injuries can be helpful*”
The impact of praxis: the impact that praxis in the field of SCI research	Praxis as a movement: Praxis has become a movement in SCI research and has earned itself a reputation	• “*Praxis has evolved, like it was first a description of what RHI does, then it’s the name of a conference, and now it is this whole movement. So, I can imagine that if in this movement moving forward, there will be a need* [for] *more meetings like Praxis. And you know we might call it Praxis 2020 or whatever, but the end the meeting is not the end all and be all, right? It’s the movement, the collaboration, the ethos that brings a big value and the conference is just one part of it*” • “*So, my expectation was that the conference, I mean the discussions of the conference were going to result in the action plan. But what unexpectedly has come out of this is that they had these spin-off meetings that happened. Some that had already been planned, but some that happened as a result of Praxis*”
	Fostered lasting consumer engagement: Praxis has resulted in greater, ongoing initiatives to engage consumers	• “*And the North American SCI Consortium that came out of* [it]*, that I feel is one tangible thing that has come out*” • “*I think* [we] *were pretty successful at getting consumers there. I think* [the sub-meeting at praxis for consumers] *was successful, and I think that has been the start of a lot of joint effort nationally and internationally with the consumer group, which is part of the Praxis action plans. I think that’s been good*”

Headings are set in bold

In terms of adoption, organisers became involved with Praxis 2016 as it aligned with their personal and/or professional interests. While organisers adopted the Praxis 2016 conference, they perceived some hesitancy on behalf of stakeholders to adopt the non-traditional and interactive format. Further, the organisers said they found it was difficult to convince stakeholders of the value of the conference’s innovative format.

Regarding implementation, high-level leadership within RHI and professional conference facilitators were brought in to facilitate the successful development implementation of the conference. Almost all organisers indicated that strong leadership and the facilitators were essential to the planning and delivery of Praxis 2016. Organisers aimed to ensure adults living with SCI were represented at every table and RHI hosted an event before the conference to help unite and empower adults with SCI to share their opinions at the conference. However, the non-traditional format did create challenges while planning Praxis 2016. The goals and format of the conference developed iteratively, which resulted in delays in the development of the conference programme and the selection of speakers. These delays were particularly challenging in terms of personnel and resources as many individuals had to work long hours to host Praxis 2016. RHI staff indicated that they felt burnt out after the conference. Organisers indicated that staff burnout and lack of a clear post-Praxis 2016 plan likely led to significant delays in the delivery of the Praxis report and action plan. Overall, organisers indicated that the conference exceeded their expectations and organisers were pleased with the non-traditional and collaborative format of the conference. Organisers were particularly pleased with the representation of adults with SCI at Praxis 2016.

In terms of setting-level maintenance, PAC and staff members felt that Praxis 2016 had become a movement in-and-of-itself that encouraged SCI research to focus on translation, and they highlighted the importance of building on the momentum established at Praxis. Members highlighted the need for continued leadership and face-to-face meetings to develop and build on short- and long-term goals identified through Praxis 2016. Of particular note, the involvement of adults with SCI in creating and implementing solutions to overcome the KT gaps was seen as an important and lasting lesson from Praxis. Members felt it was important to have efforts to continue to support and foster the engagement initiatives specific to adults with SCI such as the recent North American Spinal Cord Injury Consumer Consortium (http://www.themiamiproject.org/nascic-2017/).

### Fidelity assessment

In sessions 1–3, the majority of session goals were completed (minimum 50%, maximum 86%). In session 4, the presentation and panel discussion goals were met, but the working table was canceled due to time restraints. While the facilitator consistently achieved the outlined goals, there was variability in speakers’ ability to achieve the outlined tasks and goals. Additional file 4 outlines the percentage of session goals that were completed as intended.

### Evaluation team notes

In total, 313 implementation-based comments were extracted from the evaluation team’s notes. Key themes and definitions derived from evaluator notes as well as illustrative quotes are outlined in Table 4. Themes related to presenters and panel qualities, audience engagement and facilitation, and accessibility and engagement of adults with SCI. The majority of evaluator comments related to speakers and panels. While evaluators observed that speakers and panels had challenges adhering to the pre-defined speaker notes or goals and used jargon, presenters were generally able to foster audience engagement and present content clearly. Success stories and personal experiences were not always shared; however, evaluators noted that these stories seemed to add depth, motivation and hope for future solutions. Evaluators noted that the facilitators appeared to foster the organisation and flow of the conference and audience participation. The facilitators encouraged the audience to interact and discuss topics even when differing opinions arose, they provided clear summaries and were flexible when challenges, such as time restraints, arose. However, the audience did seem distracted at points and our reach data suggests attendance at the conference dwindled on day 2 (Table 4). Finally, evaluators noted concerns related to accessibility and the engagement and inclusion of adults with SCI. While care was taken to ensure the conference was accessible to people with SCI, the evaluators felt that adults with SCI still faced barriers to participation in all conference activities and that there was a lack of representation of adults with SCI on panels after the opening session.

**Table 4:** Themes derived from evaluator quotes

Theme	Exemplar quote from evaluator
Presenter and panel qualities	
• Fostering audience engagement: presenter spoke in a manner that seemed to engage the audience in the topic	“*Great speaker, engaging*” “*Interactive presentation*”
• Sharing personal experiences and success stories: presenter shared a personal or lived experience or success story which seemed to help convey their message or foster hope	“*Gives perspective for those without SCI, very personal explanation, many personal images*” “*Stroke* [example] *fit very well and helped give ideas and hope*” “[Some of the] *best speakers were not researchers*”
• Clear and easy to understand: the presenter’s language and/or slides were clear and easy to understand	“*Good indicator of barriers and clear solutions*” “*Clear, to the point*”
• Lacking clarity: the presenter or the slides lacked clarity, making it more difficult to understand	“*Purpose of talk was not clear*” “*Lots of jargon*”
• Adhering to speaker notes and goals: the presenter or panel followed the programme outline and covered the topics outlined	“*Covers the* [outlined] *lessons learned*” “*Followed the outline closely*” “*Good debate and conversation between panellists*”
• Not adhering to speaking notes and/or goals: the presenter or panel missed certain speaking notes and/or goals during their presentation	“*Didn’t mention any* [goals] *specifically*” “*Didn’t think this was super well covered*” “*Not so much a discussion in the panel*”
Audience Engagement and Facilitation	
• Encouraging interaction: facilitators’ ability to make the audience to be engaged and ask questions and participate during the conference	“*Encourages interaction at tables*” “*Talks about interactive participation – encouraged*”
• Providing clear summaries: facilitators’ clear summaries supported the flow of the conference	“*Program overview good*” “*Good summary of overcoming* [challenges]”
• Flexibility of facilitators: facilitators’ ability to adjust to unexpected changes helped to ensure the conference continued to flow	“*Microphone was not working well – quiet, fixed at some point*” “*Q&A period immediately after speaker*” “*Clearly explains how the program and the working group will function*”
• Distracted audience: tables and attendees were distracted at certain points throughout the conference; attendance seemed to drop-off on day 2	“*Many people on phones*” “*People texting and on computers, fidgeting*” “*Five empty tables on day 2*”
Accessibility and Engagement of the spinal chord injury (SCI) community	
• Some elements presented barriers for those with SCI: certain aspects of the conference were not accessible to individuals with SCI	“*Water kept in middle of table is inaccessible*” “*Kept saying ‘stand up’* [not really good for this conference]”
• Missing diversity on the panel: evaluators noted that members of the SCI community were rarely asked to present or be on panels	“*Why are there no consumers on the expert panel?*”

### Working table observation

JTOG scores were high across the working tables (mean 46.7, SD 4.07; minimum 42, maximum 54). These data potentially indicate that the composition of tables fostered teamwork among attendees.

## Discussion

To our knowledge, this evaluation was the first to examine the setting-level impact of a large-scale KT conference such as Praxis 2016 using the RE-AIM framework. At the setting level, both speakers and organisers adopted the Praxis model and the conference was implemented as intended. However, there were delays in post-Praxis activities and efforts were taken to ensure that Praxis 2016 impacts the SCI field long term. Broadly, our findings can be used by other groups to improve future efforts to develop large-scale KT conference solutions for translating research into practice. Key findings and recommendations in terms of adoption, implementation and maintenance are unpacked below.

RHI staff were able to establish a PAC and recruit speakers to attend and adopt the alternative conference format of Praxis 2016. However, this alternative format had benefits but also presented challenges. The hesitancy of some stakeholders and speakers to adopt the new format may explain the poor adherence to some of the implementation metrics. For example, some speakers used traditional methods of presentations in terms of length, content and style, which did not align with implementation goals of Praxis. Hesitancy to adopt alternative formats and innovations is not uncommon, as according to Rogers’ Diffusion of Innovations Theory [13], it takes time for a social system to adopt new innovations. Given this was the inaugural Praxis conference, it is likely that speakers who adhered to the implementation goals were either closely connected to RHI or vested in developing solutions to ensuring SCI research was used in practice (i.e. innovators or early adopters). If the results and movement of Praxis 2016 continue to be shared positively among SCI stakeholders, it is hoped that future iterations of this conference will see higher rates of adoption among SCI stakeholders and speakers [13]. Based on these findings, it is recommended that future KT initiatives that aim to adopt non-traditional methods work closely with stakeholders to create ownership over the methods and select individuals with established relationships with the organisation developing the initiative.

Regarding the implementation, Praxis 2016 was overall delivered as intended, with only minor deviations such as the delay in some deliverables (e.g. Action Plan) and on-site planning change (e.g. working table session 4 canceled). The impact achieved by Praxis 2016 did require extensive effort and resources from RHI staff members. Across all data sources, several key aspects related to Praxis 2016’s implementation were highlighted, including the interactive format, the value of facilitation and the value of the voice of adults with SCI. Both attendees and organisers as well as the evaluation team commented on the value of the interactive format and working tables as a strength of the Praxis 2016 format. Indeed, research has indicated that bringing together diverse opinions and fostering interdisciplinary teamwork can be valuable for KT [14, 15]. Likewise, hiring trained and professional facilitators to encourage discussion and interaction was seen as an important and valuable asset to Praxis 2016. In particular, it is recommended that organisers of future events do not underestimate the value of facilitation and bringing together diverse groups in face-to-face interactions.

An ongoing and important implementation theme was the importance of engaging the SCI community both within the Praxis 2016 initiative as well as in research and implementation more broadly. While Praxis 2016 aimed to ensure accessibility, and include people with SCI, our data indicated that further efforts are needed to foster and champion the voices of people with SCI within the translational process and at events such as Praxis. This finding aligned with calls within the SCI community for there to be “*no research about us, without us*” [16] and research funders’ emphasis on utilising and enhancing participatory and integrated KT approaches [14, 17–19]. Given organisers’ enthusiastic support for initiatives that engage adults with SCI, such as the recent North American Spinal Cord Injury Consumer Consortium (http://www.themiamiproject.org/nascic-2017/), it is anticipated that future events will have greater ownership and involvement of the SCI community throughout all phases of the event (i.e. planning, implementation and evaluation). Given these findings, it is recommended that organisers of future large-scale KT initiatives aim to partner with people with lived experience early and often throughout the planning, implementation and evaluation of their initiatives.

Findings related to the domain of maintenance begin to highlight future directions for RHI and initiatives that aim to close the gap between research and practice. While PAC and RHI staff members viewed Praxis 2016 as a movement that needs to be fostered, attendees were less clear about the long-term impact of Praxis. In particular, attendees were unsure as to whether solutions to the KT gaps between research and practice could be feasibly implemented. As the Praxis 2016 conference points out, closing the gap between research and practice is challenging. Our data suggested that ensuring the long-term impact of Praxis 2016 will require continued and persistent leadership from organisations such as RHI, as well as continued engagement from a diverse group of stakeholders, which includes people with SCI. Given this finding, it is recommended that future large-scale and event-based KT initiatives plan for, communicate about, and invest funds and personnel in post-event activities that support long-term impact.

Our evaluation methods have several strengths and limitations. In terms of strengths, the RE-AIM framework guided our evaluation, which allowed us to examine the impact of Praxis 2016 based on several perspectives and criteria. Our evaluation highlighted the value of using both qualitative and quantitative data to examine the RE-AIM domains and showed the value of triangulation for investigating the impact of complex initiatives such as Praxis. That being said, there are some limitations to our approach. In particular, fidelity checks and evaluator comments were completed in real-time rather than using recordings. Audio- and video-recordings are considered the gold standard for assessing fidelity [20]. However, the organisers did not want the tables or sessions recorded. Our team had to be embedded within the working tables and conference to complete the evaluation. While efforts were made to declare biases and maintain impartial, our findings were limited by the perceptions of the individual evaluators. Finally, our evaluation was commissioned 1 month prior to the Praxis conference. Integration of the evaluation team early in the development of Praxis 2016 may have led to additional insights.

## Conclusions

Overall, our findings indicated that RHI achieved its goals in terms of implementing Praxis 2016. Praxis 2016 was adopted by staff and stakeholders, it was broadly implemented as intended, and data indicate that Praxis 2016 has the potential to have long-term impacts in the field of SCI research and practice. This evaluation provided several lessons learned that can help to foster efforts by RHI and other groups to develop future solution-focused conferences.

## Additional files

Additional file 1: Detailed description of Praxis and evaluation procedures. (DOCX 15 kb)

Additional file 2: Interview guides. (DOCX 33 kb)

Additional file 3: Fidelity tool. (DOCX 100 kb)

Additional file 4: Implementation goals. (DOCX 19 kb)

## Abbreviations

JTOG: Jefferson Teamwork Observation Guide
KT: knowledge translation
PAC: Programme Advisory Committee
RE-AIM: reach, evaluation, adoption, implementation, maintenance
RHI: Rick Hansen Institute
SCI: spinal cord injury
