# Supplementary material for: RE-AIMing conferences: evaluating the adoption, implementation and maintenance of the Rick Hansen Institute’s Praxis 2016
Source: Health Res Policy Syst. 2019 Apr 11;17:39. doi: 10.1186/s12961-019-0434-1 (PMC6458740; doi:10.1186/s12961-019-0434-1)
Supplement: Supplementary file 1 — Detailed description of Praxis and evaluation procedures. (DOCX 15 kb) [file 12961_2019_434_MOESM1_ESM.docx]

**Supplementary File 1: Detailed Description of Praxis and Evaluation Procedures**

**Praxis 2016**

Praxis 2016 consisted of a three-day conference held in Vancouver, BC (April 25-27, 2016). The conference program was developed by RHI staff, members of the program advisory committee (PAC), and facilitators from a professional consulting company that specializes in facilitating meetings (see <http://www.strachan-tomlinson.com/>). As a whole the conference aimed to focus on developing solutions for closing the knowledge translation gaps between bench-to-bedside and bedside-to-real world application in SCI research. Day 1 consisted of an Opening Plenary Session and Welcome Reception. The Plenary Session included welcoming remarks from a variety of SCI stakeholders including researchers, clinicians, RHI staff, and people with SCI. The remainder of the conference centered around four key topics:

1. Product Development and Delivery – Challenges and Solutions in Device, Drug and Cellular Therapies.
2. Pre-Clinical and Clinical Trials of Regeneration and Repair in SCI
3. Bringing Knowledge into Clinical Practices
4. Financial Viability – Uncertain Markets, Investors, Industry, IP and Insurance-Product Development and Reimbursement

The conference aimed to follow the same format to address all four topics, with topics 1 and 2 being presented on Day 2 and topics 3 and 4 being presented on Day 3. All topics were first presented by 3-4 expert speakers per topic area. Rather than following a traditional conference style, speakers were asked to outline their translational lessons learned. Speakers were then asked to engage in a panel discussion about their lessons learned which was moderated by the conference facilitators. Finally, a working table session was held in which attendees worked in groups of 10 to develop solutions to the barriers outlined by the speakers. Attendees were asked to sit at assigned tables and organizers aimed to ensure tables included a diverse group of stakeholders. After the conference, the facilitators then worked in partnership with RHI to synthesize notes from the working tables and develop the Praxis Conference Report. The conference ended with a closing session in which session chairs summarized the learnings, conclusions, recommendations and the next steps regarding the development of a strategy-based report to guide action after the conference. Further description of the conference process, agenda and action plan^8^ are outlined in a manuscript authored by RHI^6^ and its partners and are available through the RHI website.

**Evaluation Procedures**

Setting-level data were obtained from five different sources: 1) semi-structured interviews with attendees; 2) semi-structured interviews with organizers, referring to RHI staff and PAC; 3) fidelity assessment; 4) the evaluation team’s notes; and 5) observations of working table sessions. Cost-effectiveness data were not available. Given this project is a program evaluation, ethics approval was not required; however, informed consent was obtained from all interview participants and raw data were only accessed by members of the evaluation team. Procedures associated with data collection and data analyses for each source are outlined below.

Attendees of the conference were invited to participate in a post-conference telephone interview. Convenience sampling was used; however, every effort was made to ensure a diverse group of attendees was interviewed (i.e. representation from individuals with SCI, KT specialists, researchers, industry partners, SCI community, clinicians, and policymakers). All interviews were conducted within three months post-Praxis by one evaluator (JB). A semi-structured interview guide was created specifically for attendees, and was informed by the RE-AIM framework^9^ and focused on participants’ experiences and opinions about the implementation and impact of Praxis (see Supplementary File 1). However, the interviewer was flexible and deviated from the guide to allow interviewees to focus on aspects of their experience that was important to them.

RHI staff and members of the program advisory committee involved in implementing Praxis were invited to participate in a telephone interview. A separate semi-structured interview guide was created for organizers, and was also informed by the RE-AIM framework^9^. It focused on organizers’ experiences and opinions about planning and implementing Praxis as well as their thoughts on the long-term impacts and outcomes of Praxis (see Supplementary File 2). Again, the interviewers were flexible and deviated from the guide to allow interviewees to focus on aspects of their experience that was important to them. To examine organizers’ opinions about post-Praxis activities, interviews were conducted by one of the two lead evaluators (SS and HG) within the month that followed the internal release of the Praxis Action Plan among RHI staff and the Program Advisory Committee (July 2017)^8^.

The evaluation team designed a fidelity assessment tool based on the conference agenda and specific goals for speakers, panel discussions, and working table sessions (provided by the RHI before the conference). Seven independent evaluators attended the conference and used the structured, checklist-based assessment tool to evaluate if 1) the key goals achieved during the conference (e.g. presenter outlined lessons learned), and 2) estimated duration of each aspect of the conference in minutes (e.g. working table session length). A copy of the fidelity assessment tool is provided in Supplementary File 3.

Seven members of the evaluation team attended all components of the Praxis 2016 conference. In addition to completing the fidelity assessment tool, each evaluation team member observed and took notes on the implementation of Praxis 2016.

Finally, team members were embedded randomly in different working tables. During these sessions, evaluators assessed how the working table collaborated using a modified version of the Jefferson Teamwork Observation Guide ^12^ (JTOG; See Supplementary File 3). The 14-item guide contained the identifiable competencies of a well-functioning team: values and ethics, roles and responsibilities, communication, teamwork and leadership. The JTOG was developed from expert and student feedback in the area of interprofessional education and has demonstrated sufficient reliability and validity to assess the behaviour of teams in practice.
