# Supplementary material for: RE-AIMing conferences: evaluating the adoption, implementation and maintenance of the Rick Hansen Institute’s Praxis 2016
Source: Health Res Policy Syst. 2019 Apr 11;17:39. doi: 10.1186/s12961-019-0434-1 (PMC6458740; doi:10.1186/s12961-019-0434-1)
Supplement: Supplementary file 2 — Interview guides. (DOCX 33 kb) [file 12961_2019_434_MOESM2_ESM.docx]

**Interview Guide – Praxis 2016**

**Rick Hansen Institute Staff and Praxis 2016 Program Advisory Committee (PAC)**

**Pre-interview Routine**

- Explain the purpose of the interview
- Obtain consent to interview
- Obtain consent to audio-record the interview
- Demographic Questions
  - What is your date of birth? What is your gender? What level of education do you have? What is your current role in your work place?

**Consent Script**

**Introduction**:

Hello. I’m Dr. Heather Gainforth/Dr. Shane Sweet*.* I am conducting interviews with individuals who have been integral to the development of Praxis 2016.

We will be using these interviews to examine the process used to develop Praxis, the challenges faced by the team, solutions implemented to overcome these challenges, lessons learned and recommendations for the future.

The interviews a part of an independent evaluation of the Praxis conference funded by the Rick Hansen Institute. The evaluation is being conducted by Heather Gainforth from UBC and Shane Sweet from McGill University.

**Evaluation procedures**:

I’m inviting you to do a one-on-one interview that will take about 40 – 60 minutes. I will ask you questions regarding your involvement in the development of Praxis 2016.

**Risks**:

It is not likely that there will be any serious harms or discomforts associated with the interview. There are no known physical, psychological, economic or social risks associated with this evaluation. You should not feel obligated to answer any material or participate in anything that you find objectionable or that makes you feel uncomfortable. You may also stop the interview at any time.

**Benefits:**

Your participation in this evaluation will allow for a better understanding and a further debriefing of Praxis 2016. Your participation will also allow for future conferences to be improved.

I will keep the information you tell me during the interview confidential. Information that could identify you will not be published or shared beyond the research team unless we have your permission. Any data from this research which will be shared or published will be the combined data of all participants. That means it will be reported for the whole group not for individual persons*.*

**Voluntary participation**:

- Your participation in this evaluation is voluntary.
- You can decide to stop at any time, even part-way through the interview for whatever reason.
- If you decide to stop participating, there will be no consequences to you.
- If you decide to stop we will ask you how you would like us to handle the data collected up to that point.
- This could include returning it to you, destroying it or using the data collected up to that point.
- If you do not want to answer some of the questions you do not have to, but you can still be in the evaluation.
- If you have any questions about this evaluation or would like more information you can email Heather Gainforth at heather.gainforth@ubc.ca

**Consent questions: - Start Recorder**

- Do you have any questions or would like any additional details?
- Do you agree to participate in interview knowing that you can withdraw at any point with no consequences to you?
- Do you consent to having this interview audio recorded?

**Interview Guide**

1. What was your role and involvement in Praxis 2016?

- Probe: Why did you decide to join the Praxis team (for RHI) or the PAC?
- Probe: What impact do you believe you had on Praxis 2016? (developing, running, attending)

1. In the planning phases, what impact did you anticipate Praxis 2016 would have?

- Probe: To what extent did Praxis meet (or didn’t meet) your anticipated impacts?

1. What were the positive and negative impacts of Praxis? (Probe for specific examples during and after the conference, e.g., Please describe a specific example you noticed during Praxis)

- Probe: What do you believe will be the long-term impact of Praxis on attendees, the SCI community, and for RHI?

1. In reflecting about Praxis, what went well (not well) during the planning and running of Praxis?

- Probe: What was both easy and difficult in implementing Praxis?

***We will now ask a few specific questions about Praxis. If they do not apply to you, please let us know.***

1. How did you decide who was invited to Praxis 2016?

- Probe: Methods used to make decisions?
- Probe: Recommendations for the future?

1. What was the process to select speakers?

- Probe: What, if any, challenges did you experience?

1. What was the process to find sponsors?

- Probe: What, if any, challenges did you experience?

1. What are the main impacts and challenges of the proposed Praxis 2016 action plan?

***We have a few final questions about you for our records:***

1. What is your date of birth? DD/MM/YYYY
2. What is your highest level of education?
3. What gender do you identify as?
4. What is your current role in your workplace?

**Interview Guide – Praxis 2016**

**Rick Hansen Institute Staff and Praxis 2016 Program Advisory Committee (PAC)**

**Pre-interview Routine**

- Explain the purpose of the interview
- Obtain consent to interview
- Obtain consent to audio-record the interview
- Demographic Questions
  - What is your date of birth? What is your gender? What level of education do you have? What is your current role in your work place?

**Consent Script**

**Introduction**:

Hello. I’m Dr. Heather Gainforth/Dr. Shane Sweet*.* I am conducting interviews with individuals who have been integral to the development of Praxis 2016.

We will be using these interviews to examine the process used to develop Praxis, the challenges faced by the team, solutions implemented to overcome these challenges, lessons learned and recommendations for the future.

The interviews a part of an independent evaluation of the Praxis conference funded by the Rick Hansen Institute. The evaluation is being conducted by Heather Gainforth from UBC and Shane Sweet from McGill University.

**Evaluation procedures**:

I’m inviting you to do a one-on-one interview that will take about 40 – 60 minutes. I will ask you questions regarding your involvement in the development of Praxis 2016.

**Risks**:

It is not likely that there will be any serious harms or discomforts associated with the interview. There are no known physical, psychological, economic or social risks associated with this evaluation. You should not feel obligated to answer any material or participate in anything that you find objectionable or that makes you feel uncomfortable. You may also stop the interview at any time.

**Benefits:**

Your participation in this evaluation will allow for a better understanding and a further debriefing of Praxis 2016. Your participation will also allow for future conferences to be improved.

I will keep the information you tell me during the interview confidential. Information that could identify you will not be published or shared beyond the research team unless we have your permission. Any data from this research which will be shared or published will be the combined data of all participants. That means it will be reported for the whole group not for individual persons*.*

**Voluntary participation**:

- Your participation in this evaluation is voluntary.
- You can decide to stop at any time, even part-way through the interview for whatever reason.
- If you decide to stop participating, there will be no consequences to you.
- If you decide to stop we will ask you how you would like us to handle the data collected up to that point.
- This could include returning it to you, destroying it or using the data collected up to that point.
- If you do not want to answer some of the questions you do not have to, but you can still be in the evaluation.
- If you have any questions about this evaluation or would like more information you can email Heather Gainforth at heather.gainforth@ubc.ca

**Consent questions: - Start Recorder**

- Do you have any questions or would like any additional details?
- Do you agree to participate in interview knowing that you can withdraw at any point with no consequences to you?
- Do you consent to having this interview audio recorded?

**Interview Guide**

1. What was your role and involvement in Praxis 2016?

- Probe: Why did you decide to join the Praxis team (for RHI) or the PAC?
- Probe: What impact do you believe you had on Praxis 2016? (developing, running, attending)

1. In the planning phases, what impact did you anticipate Praxis 2016 would have?

- Probe: To what extent did Praxis meet (or didn’t meet) your anticipated impacts?

1. What were the positive and negative impacts of Praxis? (Probe for specific examples during and after the conference, e.g., Please describe a specific example you noticed during Praxis)

- Probe: What do you believe will be the long-term impact of Praxis on attendees, the SCI community, and for RHI?

1. In reflecting about Praxis, what went well (not well) during the planning and running of Praxis?

- Probe: What was both easy and difficult in implementing Praxis?

***We will now ask a few specific questions about Praxis. If they do not apply to you, please let us know.***

1. How did you decide who was invited to Praxis 2016?

- Probe: Methods used to make decisions?
- Probe: Recommendations for the future?

1. What was the process to select speakers?

- Probe: What, if any, challenges did you experience?

1. What was the process to find sponsors?

- Probe: What, if any, challenges did you experience?

1. What are the main impacts and challenges of the proposed Praxis 2016 action plan?

***We have a few final questions about you for our records:***

1. What is your date of birth? DD/MM/YYYY
2. What is your highest level of education?
3. What gender do you identify as?
4. What is your current role in your workplace?
