# Supplementary material for: RE-AIMing conferences: evaluating the adoption, implementation and maintenance of the Rick Hansen Institute’s Praxis 2016
Source: Health Res Policy Syst. 2019 Apr 11;17:39. doi: 10.1186/s12961-019-0434-1 (PMC6458740; doi:10.1186/s12961-019-0434-1)
Supplement: Supplementary file 3 — Fidelity tool. (DOCX 100 kb) [file 12961_2019_434_MOESM3_ESM.docx]

| **Background Information** | | |
| --- | --- | --- |
| **Session description**: The opening plenary session sets the tone for the conference purpose: to convene stakeholders from across the SCI continuum as well as other fields to build awareness, share experiences, and workshop practical solutions to address the two translational “Valleys of Death”. Praxis 2016 is a platform for collaboration and will challenge participants to work collectively to identify actionable solutions. | | |
| **Key Goals** | | |
| *Tasks* | *Speaker(s)* | *Comments* |
| - Participants welcomed to the first Praxis |  |  |
| - A range of perspectives to sharpen the focus on the conference objectives |  |  |
| - Participants invited to take part in the poster presentation |  |  |
| - Encourages introductions at discussion tables |  |  |
| **Bill Barrable, RHI (______ minutes)** | | |
| ***Tasks*** | *Total Time* | *Comments* |
| - Discuses challenges/barriers they have and are facing in their work and/or personal experiences |  |  |
| - Discusses solutions to challenges barriers |  |  |
| - Discusses how their learnings can be leveraged into discussion among participants regarding concrete actions |  |  |
| Take home points: | | |
| **Graham Creasey, Praxis 2016 Chair (______ minutes)** | | |
| ***Tasks*** | *Total Time* | *Comments* |
| - Discuses challenges/barriers they have and are facing in their work and/or personal experiences |  |  |
| - Discusses solutions to challenges barriers |  |  |
| - Discusses how their learnings can be leveraged into discussion among participants regarding concrete actions |  |  |
| Take home points: | | |
| **Hon. Carla Qualtrough**, **Federal Minister of Sport and Persons with Disabilities *(______ minutes)*** | | |
| ***Tasks*** | *Total Time* | *Comments* |
| - Discuses challenges/barriers they have and are facing in their work and/or personal experiences |  |  |
| - Discusses solutions to challenges barriers |  |  |
| - Discusses how their learnings can be leveraged into discussion among participants regarding concrete actions |  |  |
| Take home points: | | |
| **Eric Marcotte**, **Canadian Institutes of Health Research (______ minutes)** | | |
| ***Tasks*** | *Total Time* | *Comments* |
| - Discuses challenges/barriers they have and are facing in their work and/or personal experiences |  |  |
| - Discusses solutions to challenges barriers |  |  |
| - Discusses how their learnings can be leveraged into discussion among participants regarding concrete actions |  |  |
| Take home points: | | |
| **Rick Hansen**, **Canadian Institutes of Health Research (______ minutes)** | | |
| ***Tasks*** | *Total Time* | *Comments* |
| - Discuses challenges/barriers they have and are facing in their work and/or personal experiences |  |  |
| - Discusses solutions to challenges barriers |  |  |
| - Discusses how their learnings can be leveraged into discussion among participants regarding concrete actions |  |  |
| Take home points: | | |
| **Jennifer French**, **Neurotech Network (______ minutes)** | | |
| ***Tasks*** | *Total Time* | *Comments* |
| - Discuses challenges/barriers they have and are facing in their work and/or personal experiences |  |  |
| - Discusses solutions to challenges barriers |  |  |
| - Discusses how their learnings can be leveraged into discussion among participants regarding concrete actions |  |  |
| Take home points: | | |
| **Kim Anderson-Erisman**, **Miami Project to Cure Paralysis (______ minutes)** | | |
| ***Tasks*** | *Total Time* | *Comments* |
| - Discuses challenges/barriers they have and are facing in their work and/or personal experiences |  |  |
| - Discusses solutions to challenges barriers |  |  |
| - Discusses how their learnings can be leveraged into discussion among participants regarding concrete actions |  |  |
| Take home points: | | |
| **Francesco Marincola, Sidra Medical & Research Center - Sponsored by BC Cancer Agency (______ minutes)** | | |
| ***Tasks*** | *Total Time* | *Comments* |
| - Discuses challenges/barriers they have and are facing in their work and/or personal experiences |  |  |
| - Discusses solutions to challenges barriers |  |  |
| - Discusses how their learnings can be leveraged into discussion among participants regarding concrete actions |  |  |
| Take home points: | | |

**LESSONS LEARNED PANEL (Start Time:__________; End Time:_____________)**

| **Background Information** | | |
| --- | --- | --- |
| Description: The development and delivery of new medical devices, drugs and cellular therapies is fraught with obstacles and barriers beyond the demonstration of efficacy. Practical strategies and significant financial resources are required to get a product into clinical use and commercial viability. | | |
| **Facilitator Discussion ( ______ minutes); Presenter Name:_________________** | | |
| *Tasks* | *Total Time* | *Comments* |
| - Sets expectations for and flow of Praxis |  | ^1^ |
| - Discusses handout |  | ^2^ |
| - Discusses guidelines for working together |  | ^3^ |
| - Introduce session and panel |  | ^4^ |
| **Lessons Learned Panel ( ______ minutes)** |  | ^5^ |
| ***Hunter Peckham, Donnell Institute Professor of Biomedical Engineering*** | *Total Time* | *Comments* |
| - Provides 2-3 significant challenges or barriers to implementation |  | ^6^ |
| - Provides key learning/advice for next time |  | ^7^ |
| - Does NOT use powerpoint   - Number of slides used _________ |  | ^8^ |
| *Speaking Notes* |  |  |
| - Overview of both valleys with focus on medical devices - Receiving approval and traversing both valleys - Tangible learnings and intangible learning examples shared - Moving innovation into the world-wide sphere |  | ^9^  ^10^  ^11^  ^12^ |
| Take home points: | | |
| ***Andrew Blight, Chief Scientific Officer, Acorda Therapeutics*** | *Total Time* | *Comments* |
| - Provides 2-3 significant challenges or barriers to implementation |  | ^13^ |
| - Provides key learning/advice for next time |  | ^14^ |
| - Does NOT use powerpoint   - Number of slides used _________ |  | ^15^ |
| *Speaking Notes* |  |  |
| - Getting drugs into clinical trials - Universal problem – make link to session 2 and session 1 - Development of standards for product solutions - Development of right instruments - Tools to develop right instruments |  | ^16^  ^17^  ^18^  ^19^  ^20^ |
| Take home points: | | |
| ***Edward Wirth, Chief Medical Officer, Asterias Biotherapeutics*** | *Total Time* | *Comments* |
| - Provides 2-3 significant challenges or barriers to implementation |  | ^21^ |
| - Provides key learning/advice for next time |  | ^22^ |
| - Does NOT use powerpoint   - Number of slides used _________ |  | ^23^ |
| *Speaking Notes* |  |  |
| - Lessons learned from commercial stem cell study - Issues with cells - Examples of high profile failures in other countries - Geron Corporation example - SCOPE example - Discuss take-away points |  | ^24^  ^25^  ^26^  ^27^  ^28^ |
| Take home points: |  |  |
| ***Megan Moynahan, Executive Director, Institute for Functional Restoration*** | *Total Time* | *Comments* |
| - Provides 2-3 significant challenges or barriers to implementation |  | ^29^ |
| - Provides key learning/advice for next time |  | ^30^ |
| - Does NOT use powerpoint   - Number of slides used _________ |  | ^31^ |
| *Speaking Notes* |  |  |
| - How to keep drug/device sustainable in SCI market - Interesting emerging models in commercialization - Corporate social responsibility - De-risk business model - Collaborative multi-pronged attack - Direct consumer marketing - Work with individuals with advocacy skills - Make a link with session 4 |  | ^32^  ^33^  ^34^  ^35^  ^36^  ^37^  ^38^ |
| Take home points: |  |  |
| **Panel Interaction ( ______ minutes)** |  |  |
| *Tasks* | *Total Time* | *Comments* |
| - Panelists discuss key points/remarks of other speakers |  | ^39^ |
| - Panelists discuss areas of agreement |  | ^40^ |
| - Panelists discuss areas of disagreement |  | ^41^ |
| - Panelists discuss promising solutions (e.g. proud of) |  | ^42^ |
| - Other discussion: |  | ^43^ |

If you have additional comments for the Lessons Learned Panel, write in the box below with their comment number:

**WORKING SESSION (1 panel table; 6 randomly selected tables); (Start Time:__________; End Time:_____________)**

| **TABLE___________** | | | | |
| --- | --- | --- | --- | --- |
| **Group Members** | | | | |
| *Name* | *Role* | *Name* | | *Role* |
|  |  |  | |  |
|  |  |  | |  |
|  |  |  | |  |
|  |  |  | |  |
|  |  |  | |  |
| **Discussion (_________ minutes)** | | | | |
| *Tasks* | | *Total Time* | *Comments* | |
| - Group introduces themselves and their perspective | |  | ^44^ | |
| - The group selects two challenges that resonate with them | |  | ^45^ | |
| *Challenge 1:* | | *Total Time* | *Comments* | |
| - Discuss how the challenged has affected them personally (individual and/or others) | |  | ^46^ | |
| - Discuss how the challenged has affected them professionally (individual and/or others) | |  | ^47^ | |
| - Discuss what would real progress look like | |  | ^48^ | |
| - Discuss how to achieve progress together | |  | ^49^ | |
| - For each action, indicate individuals that need to be engaged | |  | ^50^ | |
| *Description of actions:* | | | | |
| *Challenge 2:* | | *Total Time* | *Comments* | |
| - Discuss how the challenged has affected them personally (individual and/or others) | |  | ^51^ | |
| - Discuss how the challenged has affected them professionally (individual and/or others) | |  | ^52^ | |
| - Discuss what would real progress look like | |  | ^53^ | |
| - Discuss how to achieve progress together | |  | ^54^ | |
| - For each action, indicate individuals that need to be engaged | |  | ^55^ | |
| *Description of actions:* | | | | |
| **Facilitator Discussion ( ______ minutes); Presenter Name:_________________** | | | | |
| *Tasks* | | *Total Time* | *Comments* | |
| - Each group provides the #1 action | |  | ^56^ | |
| - Each group provides who needs to be involved | |  | ^57^ | |
| - Discusses overlap and mutually supportive approaches | |  | ^58^ | |
| - Session chair adds to the discussion | |  | ^59^ | |
| **Other** | | *Total Time* | *Comments* | |
| - Note-taker provides a summary of strategies mentioned | |  | ^60^ | |
| - Facilitator summarizes solutions later in the day/next day | |  | ^61^ | |

If you have additional comments for Working Session 1, write in the box below with their comment number:

**Table Observation Guide**

At the end of each working session, please rate your level of agreement (SD = strong disagree; SA = strongly agree) to each of the following statements.

| Statement | SD |  |  | SA | N/A |
| --- | --- | --- | --- | --- | --- |
| 1. There appeared to be a leader that coordinated the discussion.   (Name of leader: ____________________) | 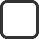 | 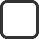 | 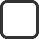 | 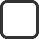 | 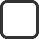 |
| 1. The leader facilitated the discussion rather than dominated it. | 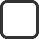 | 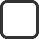 | 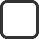 | 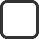 | 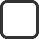 |
| 1. Members came prepared to discuss the topic from their profession-specific perspective. | 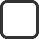 | 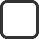 | 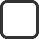 | 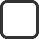 | 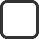 |
| 1. Members who were involved in the topic contributed to the discussion. | 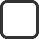 | 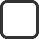 | 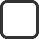 | 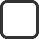 | 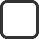 |
| 1. Discussion was distributed among all members. | 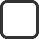 | 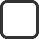 | 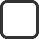 | 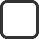 | 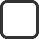 |
| 1. Members appeared to understand the roles and responsibilities of other members of the team. | 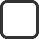 | 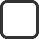 | 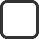 | 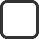 | 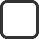 |
| 1. Members appeared to have respect, confidence, and trust in one another. | 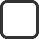 | 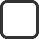 | 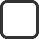 | 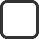 | 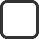 |
| 1. Members listened and paid attention to each other. | 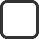 | 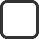 | 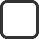 | 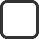 | 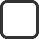 |
| 1. Members listened to and considered the input of others before pressing their own ideas. | 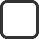 | 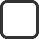 | 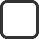 | 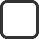 | 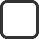 |
| 1. Members added other supporting pieces of information from their profession-specific perspective regarding the topic. | 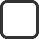 | 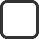 | 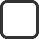 | 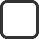 | 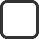 |
| 1. The opinions of members were valued by other members. | 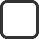 | 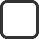 | 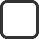 | 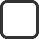 | 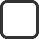 |
| 1. Members appeared to feel free to disagree openly with each other’s ideas. | 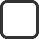 | 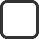 | 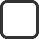 | 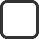 | 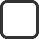 |
| 1. Members sought out opportunities to work with others on specific tasks. | 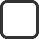 | 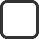 | 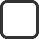 | 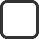 | 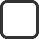 |
| 1. Members engaged in friendly interaction with one another. | 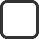 | 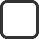 | 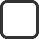 | 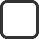 | 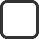 |

| ***Other Observations*** *(e.g. barriers/facilitators to participating in the discussion; lack of participation by certain members)* |
| --- |

**CASE STUDY EXPERIENCES PANEL (Start Time:__________ ; End Time:________)**

***Collect a copy of handouts given**

| **Background Information** | | |
| --- | --- | --- |
| Description: Although several SCI clinical trials for neuroprotection (protecting the spinal cord from secondary damage after injury) or to promote regeneration/restoration of spinal nerve circuitry have been undertaken and are ongoing, few have proceeded past the stage of safety and early efficacy studies and no effective treatment to restore function has been demonstrated. | | |
| **Session Chair ( ______ minutes); Presenter Name:_________________** | | |
| *Tasks* | *Total Time* | *Comments* |
| - Introduce session and panel |  | ^1^ |
| **Case Study Experiences Panel ( ______ minutes)** |  | ^2^ |
| ***Lyn Jakeman, Program Director, National Institutes of Health/National Institute of Neurological Disorders and Stroke*** | *Total Time* | *Comments* |

| - Presents a case study based on experience |  | ^3^ |
| --- | --- | --- |
| - Named 2-3 barriers in the at-a-glance handout |  | ^4^ |

| - Mentions how to overcome barriers in hindsight |  | ^5^ |
| --- | --- | --- |
| - Provides what needs to be done in future |  | ^6^ |
| - Does NOT use powerpoint   - Number of slides used _________ |  | ^7^ |
| *Speaking Notes* |  |  |
| - Why trials fail - How funders place their bets - Recommend changes to prevent future failures - Discuss robustness of pre-clinical data (animals) for clinical trials (humans) |  | ^8^  ^9^  ^10^  ^11^ |
| Take home points: | | |
| ***Brian Kwon, Canada Research Chair in Spinal Cord Injury, University of British Columbia*** | *Total Time* | *Comments* |

| - Presents a case study based on experience |  | ^12^ |
| --- | --- | --- |
| - Named 2-3 barriers in the at-a-glance handout |  | ^13^ |

| - Mentions how to overcome barriers in hindsight |  | ^14^ |
| --- | --- | --- |
| - Provides what needs to be done in future |  | ^15^ |
| - Does NOT use powerpoint   - Number of slides used _________ |  | ^16^ |
| *Speaking Notes* |  |  |
| - Key challenges - Retrospective view: implications for future studies - Animal models vs. human realities |  | ^17^  ^18^  ^19^ |
| Take home points: | | |
| ***Michelle (Shelly) Towle, Assistant Director, SCI Program, DP Clinical Contract Research*** | *Total Time* | *Comments* |

| - Presents a case study based on experience |  | ^20^ |
| --- | --- | --- |
| - Named 2-3 barriers in the at-a-glance handout |  | ^21^ |

| - Mentions how to overcome barriers in hindsight |  | ^22^ |
| --- | --- | --- |
| - Provides what needs to be done in future |  | ^23^ |
| - Does NOT use powerpoint   - Number of slides used _________ |  | ^24^ |
| *Speaking Notes* |  |  |
| - Overview of challenges specific to her case (e.g. proof of concept) - Discusses solutions that may work (e.g. protocol less arduous) |  | ^25^  ^26^  ^27^  ^28^ |
| Take home points: | | |
| **Panel Interaction ( ______ minutes)** |  |  |
| *Tasks* | *Total Time* | *Comments* |
| - Panelists what stands out with respect to future change |  | ^29^ |
| - Panelists discuss key improvements (pre clinical 🡪 clinical) |  | ^30^ |
| - Other discussion: |  | ^31^ |

If you have additional comments for the Lessons Learned Panel, write in the box below with their comment number:

**WORKING SESSION (1 panel table; 6 randomly selected tables)**

| **TABLE___________** | | | | |
| --- | --- | --- | --- | --- |
| **Group Members** | | | | |
| *Name* | *Role* | *Name* | | *Role* |
|  |  |  | |  |
|  |  |  | |  |
|  |  |  | |  |
|  |  |  | |  |
|  |  |  | |  |
| **Discussion (_________ minutes)** | | | | |
| *Tasks* | | *Total Time* | *Comments* | |
| - The group recommends strategies to initiate before undertaking clinical trials | |  | ^32^ | |
| - Discuss what would ensure higher completion rates of such trials | |  | ^33^ | |
| - Select preclinical or clinical time frame for incentives | |  | ^34^ | |
| - Discuss incentives to ensure ideas are implemented | |  | ^35^ | |
| Other Discussion: ^36^ | | | | |
| **Facilitator Discussion ( ______ minutes); Presenter Name:_________________** | | | | |
| *Tasks* | | *Total Time* | *Comments* | |
| - Chair gathers and summarizes groups recommendations | |  | ^37^ | |
| - Discusses overlap and mutually supportive approaches | |  | ^38^ | |
| **Other** | | *Total Time* | *Comments* | |
| - Note-taker provides a summary of strategies mentioned | |  | ^39^ | |
| - Facilitator summarizes solutions later in the day/next day | |  | ^40^ | |

If you have additional comments for Working Session 2, write in the box below with their comment number:

**Table Observation Guide**

At the end of each working session, please rate your level of agreement (SD = strong disagree; SA = strongly agree) to each of the following statements.

| Statement | SD |  |  | SA | N/A |
| --- | --- | --- | --- | --- | --- |
| 1. There appeared to be a leader that coordinated the discussion.   (Name of leader: ____________________) | 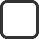 | 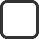 | 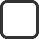 | 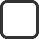 | 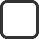 |
| 1. The leader facilitated the discussion rather than dominated it. | 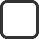 | 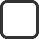 | 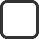 | 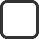 | 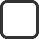 |
| 1. Members came prepared to discuss the topic from their profession-specific perspective. | 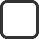 | 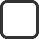 | 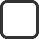 | 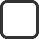 | 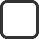 |
| 1. Members who were involved in the topic contributed to the discussion. | 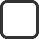 | 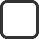 | 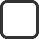 | 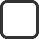 | 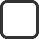 |
| 1. Discussion was distributed among all members. | 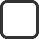 | 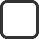 | 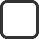 | 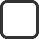 | 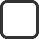 |
| 1. Members appeared to understand the roles and responsibilities of other members of the team. | 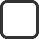 | 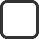 | 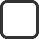 | 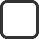 | 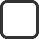 |
| 1. Members appeared to have respect, confidence, and trust in one another. |  |  |  |  |  |
| 1. Members listened and paid attention to each other. |  |  |  |  |  |
| 1. Members listened to and considered the input of others before pressing their own ideas. |  |  |  |  |  |
| 1. Members added other supporting pieces of information from their profession-specific perspective regarding the topic. |  |  |  |  |  |
| 1. The opinions of members were valued by other members. |  |  |  |  |  |
| 1. Members appeared to feel free to disagree openly with each other’s ideas. |  |  |  |  |  |
| 1. Members sought out opportunities to work with others on specific tasks. |  |  |  |  |  |
| 1. Members engaged in friendly interaction with one another. |  |  |  |  |  |

| ***Other Observations*** *(e.g. barriers/facilitators to participating in the discussion; lack of participation by certain members)* |
| --- |

**PROSPECTIVES PANEL (Start Time:__________ ; End Time:___________)**

| **Background Information** | | |
| --- | --- | --- |
| Description: Implementation of leading practices and standardization of care are challenging endeavours in a broad range of areas, including SCI. Although efficient rehabilitation interventions, standards of practice, guidelines, various clinical tools and protocols as well as approved technologies exist for numerous areas of SCI care, their implementation is often inefficient and uneven among facilities and regions. Numerous factors contribute to these gaps, including funding, policy and behaviour change issues. | | |
| **Facilitator Discussion ( ______ minutes); Presenter Name:_________________** | | |
| *Tasks* | *Total Time* | *Comments* |
| - Introduces session and panel |  | ^1^ |
| - Disucsses group results from yesterday |  | ^2^ |
| - Overview of key actions and soluations |  | ^3^ |
| - Discusses implications for today |  | ^4^ |
| - Introduce session and panel |  | ^5^ |
| **Prospectives Panel ( ______ minutes)** |  |  |
| ***Anthony Burns, Physiatrist, UHN-Toronto Rehabilitation Institute; Associate Professor, Dept of Medicine, U. of Toronto*** | *Total Time* | *Comments* |
| - Provides what works and what does not in regard to implementation |  | ^6^ |
| - Provides what needs to happen to facilitate implementation |  | ^7^ |
| - Does NOT use powerpoint   - Number of slides used:____________ |  | ^8^ |
| *Speaking Notes* |  |  |
| - Discusses the nature of rehabilitation research - Research culture vs. clinical culture - Discusses effective translation and implementation |  | ^9^  ^10^  ^11^ |
| Take home points: | | |
| ***Mark Bayley, Medical Director, Brain & Spinal Cord Rehab Program, UHN-Toronto, Rehabilitation Institute*** | *Total Time* | *Comments* |
| - Provides what works and what does not in regard to implementation |  | ^12^ |
| - Provides what needs to happen to facilitate implementation |  | ^13^ |
| - Does NOT use powerpoint   - Number of slides used: ____________ |  | ^14^ |
| *Speaking Notes* |  |  |
| - Defines whose behaviour they aim to change - Discusses what stragies do/do not work - Role of leadership and organizational culture - Discusses solutions |  | ^15^  ^16^  ^17^  ^18^ |
| Take home points: | | |
| ***Cathy Craven, Medical Lead, Brain & Spinal Cord Rehab Program, Toronto Rehabilitation Institute. Associate Professor, Department of Medicine, University of Toronto*** | *Total Time* | *Comments* |
| - Provides what works and what does not in regard to implementation |  | ^19^ |
| - Provides what needs to happen to facilitate implementation |  | ^20^ |
| - Does NOT use powerpoint   - Number of slides used |  | ^21^ |
| *Speaking Notes* |  |  |
| - Disucsses 3 key challenges in moving knowledge into practice - Illustrates lack of uptake - Discusses strength of practice-based evidence - Provides strategies to address disconnect |  | ^22^  ^23^  ^24^  ^25^ |
| Take home points: | | |
| **Panel Interaction ( ______ minutes)** |  |  |
| *Tasks* | *Total Time* | *Comments* |
| - Ask Anthony and Cathy about what they heard from Mark that can be applied to SCI |  | ^26^ |
| - Discuss what promising practices are not in general use |  | ^27^ |
| - Plenary Q&A emerges from panel discussion |  | ^28^ |
| - Other discussion: |  | ^29^ |

If you have additional comments for the Lessons Learned Panel, write in the box below with their comment number:

**WORKING SESSION (1 panel table; 6 randomly selected tables)**

| **TABLE___________** | | | | |
| --- | --- | --- | --- | --- |
| **Group Members** *(*symbols denote commitment to solving each challenge)* | | | | |
| *Name* | *Role* | *Name* | | *Role* |
|  |  |  | |  |
|  |  |  | |  |
|  |  |  | |  |
|  |  |  | |  |
|  |  |  | |  |
| **Discussion (_________ minutes)** | | | | |
| *Tasks* | | *Total Time* | *Comments* | |
| - The group selects two tools and strategies | |  | ^30^ | |
| *Tool/Strategy 1:* | | *Total Time* | *Comments* | |
| - Discuss the type of practice it would be best applied to | |  | ^31^ | |
| - Discuss vision for 2020 | |  | ^32^ | |
| - Discusses what need to be done to implement vision | |  | ^33^ | |
| - For each action, indicate key players that need to be involved | |  | ^34^ | |
| *Description of action/vision:* | | | | |
| *Tool/Strategy 2:* | | *Total Time* | *Comments* | |
| - Discuss the type of practice it would be best applied to | |  | ^35^ | |
| - Discuss vision for 2020 | |  | ^36^ | |
| - Discusses what need to be done to implement vision | |  | ^37^ | |
| - For each action, indicate key players that need to be involved | |  | ^38^ | |
| *Description of action/vision:* | | | | |
| **Facilitator Discussion ( ______ minutes); Presenter Name:_________________** | | | | |
| *Tasks* | | *Total Time* | *Comments* | |
| - Discusses similarities, differences and insights | |  | ^39^ | |
| - Session chair adds to the discussion | |  | ^40^ | |

If you have additional comments for Working Session 1, write in the box below with their comment number:

**Table Observation Guide**

At the end of each working session, please rate your level of agreement (SD = strong disagree; SA = strongly agree) to each of the following statements.

| Statement | SD |  |  | SA | N/A |
| --- | --- | --- | --- | --- | --- |
| 1. There appeared to be a leader that coordinated the discussion.   (Name of leader: ____________________) |  |  |  |  |  |
| 1. The leader facilitated the discussion rather than dominated it. |  |  |  |  |  |
| 1. Members came prepared to discuss the topic from their profession-specific perspective. |  |  |  |  |  |
| 1. Members who were involved in the topic contributed to the discussion. |  |  |  |  |  |
| 1. Discussion was distributed among all members. |  |  |  |  |  |
| 1. Members appeared to understand the roles and responsibilities of other members of the team. |  |  |  |  |  |
| 1. Members appeared to have respect, confidence, and trust in one another. |  |  |  |  |  |
| 1. Members listened and paid attention to each other. |  |  |  |  |  |
| 1. Members listened to and considered the input of others before pressing their own ideas. |  |  |  |  |  |
| 1. Members added other supporting pieces of information from their profession-specific perspective regarding the topic. |  |  |  |  |  |
| 1. The opinions of members were valued by other members. |  |  |  |  |  |
| 1. Members appeared to feel free to disagree openly with each other’s ideas. |  |  |  |  |  |
| 1. Members sought out opportunities to work with others on specific tasks. |  |  |  |  |  |
| 1. Members engaged in friendly interaction with one another. |  |  |  |  |  |

| ***Other Observations*** *(e.g. barriers/facilitators to participating in the discussion; lack of participation by certain members)* |
| --- |

**EXPERIENCE-IN-ACTION PANEL (Start Time:__________; End Time:__________)**

| **Background Information** | | |
| --- | --- | --- |
| Description: One barrier to clinical studies and commercialization of SCI-related innovations is limited financial resources for first-in-man and placebo-controlled studies for novel drugs or cell-based therapies. Testing new entities in patients requires the financial resources to support patents, GMP product manufacturing, and non-clinical safety studies in at least two species. Successful commercialization requires pivotal clinical studies that demonstrate robust functional improvement as well as value for patients to ensure reimbursement from insurance companies and government payers. Early stage investors and industry play critical roles in translating innovative ideas into commercial products. However, products related to SCI are often not considered attractive for investment due to a lack of understanding of the potential to improve patient independence and quality of life. Also, the unknown revenue potential of the target market, and uncertainty of securing payer reimbursements make financing SCI clinical studies risky. | | |
| **Facilitator Discussion ( ______ minutes); Presenter Name:_________________** | | |
| *Tasks* | *Total Time* | *Comments* |
| - Introduces session |  | ^1^ |
| - Introduces panel and topic |  | ^2^ |
| - Discusses implications for today |  | ^3^ |
| **Table Introductions ( ______ minutes)** | | |
| *Tasks* | *Total Time* | *Comments* |
| ______ /______ tables engaged in introductions (i.e. did they speak) |  | ^4^ |
| **Experience-in-Action Panel ( ______ minutes)** |  | ^5^ |
| ***Phil Tinmouth, Vice President & Head, Business Development & Alliance Management, Vertex Pharmaceuticals*** | *Total Time* | *Comments* |
| - Provides 2-3 challenges |  | ^6^ |
| - Provides primary solution to address challenges |  | ^7^ |
| - Outlines what key 1 or 2 driving forces would support the implementation of your solution |  | ^8^ |
| - Outlines 1 or 2 key restraining forces would need to be addressed and how to address them |  | ^9^ |
| - Does NOT use powerpoint   - Number of slides used____________ |  | ^10^ |
| *Speaking Notes* |  |  |
| - Discusses financial viability and the impact on patient well-being - Discusses company involvement and perspectives - Discusses value of partnership - Proposal: Introduces a proposal/solution to support translation of research towards commercial development |  | ^11^  ^12^  ^13^  ^14^  ^15^  ^16^ |
| Take home points: | | |
| ***Kathleen Marsman, Patent Agent, Borden Ladner Gervais LLP*** | *Total Time* | *Comments* |
| - Provides 2-3 challenges |  | ^17^ |
| - Provides primary solution to address challenges |  | ^18^ |
| - Outlines what key 1 or 2 driving forces would support the implementation of your solution |  | ^19^ |
| - Outlines 1 or 2 key restraining forces would need to be addressed and how to address them |  | ^20^ |
| - Does NOT use powerpoint   - Number of slides used___________ |  | ^21^ |
| *Speaking Notes* |  |  |
| - Discusses top 3 IP questions - Discusses CHIR commercialization grants - Role of IP for devices - Provides examples of how patient issues have been addressed - Proposal: support protection of IP at the appropriate time |  | ^22^  ^23^  ^24^  ^25^  ^26^ |
| Take home points: | | |
| ***Dennis Choi, Professor and Chair, Department of Neurology, Stony Brook Medicine*** | *Total Time* | *Comments* |
| - Provides 2-3 challenges |  | ^27^ |
| - Provides primary solution to address challenges |  | ^28^ |
| - Outlines what key 1 or 2 driving forces would support the implementation of your solution |  | ^29^ |
| - Outlines 1 or 2 key restraining forces would need to be addressed and how to address them |  | ^30^ |
| - Does NOT use powerpoint   - Number of slides used_________ |  | ^31^ |
| *Speaking Notes* |  |  |
| - Provides a Big phrama point of view - Discusses differences between academic approach and industry approach - Discusses challenges to be discussed - Discusses the effectiveness of biomarkers - Proposal: organized advocacy and regulatory change as well as the rising role of biotech and foundations |  | ^32^  ^33^  ^34^  ^35^  ^36^ |
| Take home points: |  |  |
| ***Ron Podraza, Co-Founder and CEO, Reimbursement Principles, Inc.*** | *Total Time* | *Comments* |
| - Provides 2-3 challenges |  | ^37^ |
| - Provides primary solution to address challenges |  | ^38^ |
| - Does NOT use powerpoint   - Number of slides used__________ |  | ^39^ |
| *Speaking Notes* |  |  |
| - Links session 1 to other sessions - Discusses principles of reimbursement and reimbursement strategy - Proposal: support commercialization in the future |  | ^40^  ^41^  ^42^ |
| Take home points: |  |  |

If you have additional comments for the Lessons Learned Panel, write in the box below with their comment number:

**WORKING SESSION (1 panel table; 6 randomly selected tables)**

| **TABLE___________** | | | | |
| --- | --- | --- | --- | --- |
| **Group Members** *(*symbols denote commitment to solving each challenge)* | | | | |
| *Name* | *Role* | *Name* | | *Role* |
| ① |  | ① | |  |
| ① |  | ① | |  |
| ① |  | ① | |  |
| ① |  | ① | |  |
| ① |  | ① | |  |
| **Discussion (_________ minutes)** | | | | |
| *Tasks* | | *Total Time* | *Comments* | |
| - The group is given the implementation they will lead | |  | ^43^ | |
| *Implementation of proposal outlined in worksheet:* | | *Total Time* | *Comments* | |
| - Discusses any clarification they have of panel regarding the proposal | |  | ^44^ | |
| - Discusses concerns around implementation of the proposal | |  | ^45^ | |
| - Discusses considerations around implementation of the proposal | |  | ^46^ | |
| **Plenary Discussion and Panel member summary ( ______ minutes); Presenter Name:_________________** | | | | |
| *Tasks* | | *Total Time* | *Comments* | |
| - Discusses questions from groups | |  | ^47^ | |
| - Discuss aspects to round out speaker’s proposals | |  | ^48^ | |
| - Session chair adds to the discussion | |  | ^49^ | |
| - Panel members invited to respond (check if they respond):   - Phil Tinmouth   - Kathleen Marsman   - Dennis Choi   - Ron Podraza | |  | ^50^  ^51^  ^52^  ^53^  ^54^ | |

If you have additional comments for Working Session 4, write in the box below with their comment number:

**Table Observation Guide**

At the end of each working session, please rate your level of agreement (SD = strong disagree; SA = strongly agree) to each of the following statements.

| Statement | SD |  |  | SA | N/A |
| --- | --- | --- | --- | --- | --- |
| 1. There appeared to be a leader that coordinated the discussion.   (Name of leader: ____________________) |  |  |  |  |  |
| 1. The leader facilitated the discussion rather than dominated it. |  |  |  |  |  |
| 1. Members came prepared to discuss the topic from their profession-specific perspective. |  |  |  |  |  |
| 1. Members who were involved in the topic contributed to the discussion. |  |  |  |  |  |
| 1. Discussion was distributed among all members. |  |  |  |  |  |
| 1. Members appeared to understand the roles and responsibilities of other members of the team. |  |  |  |  |  |
| 1. Members appeared to have respect, confidence, and trust in one another. |  |  |  |  |  |
| 1. Members listened and paid attention to each other. |  |  |  |  |  |
| 1. Members listened to and considered the input of others before pressing their own ideas. |  |  |  |  |  |
| 1. Members added other supporting pieces of information from their profession-specific perspective regarding the topic. |  |  |  |  |  |
| 1. The opinions of members were valued by other members. |  |  |  |  |  |
| 1. Members appeared to feel free to disagree openly with each other’s ideas. |  |  |  |  |  |
| 1. Members sought out opportunities to work with others on specific tasks. |  |  |  |  |  |
| 1. Members engaged in friendly interaction with one another. |  |  |  |  |  |

| ***Other Observations*** *(e.g. barriers/facilitators to participating in the discussion; lack of participation by certain members)* |
| --- |

| **Background Information** | | |
| --- | --- | --- |
| **Session description**: This session provides the transition between the conference and the development of an action plan. The session chairs will be listening and reflecting and discussing with each other throughout the conference essential actions for crossing the two Valleys of Death, and how to present them in this session.  Each speaker has 5 minutes to make 2 or 3 points related to insights and recommendations for action. Each speaker will close with “Here is what I am prepared to do to support addressing the gaps discussed in relation to Valleys 1 and 2 throughout the conference.” These personal commitments need to be about what is do-able in an everyday busy life. The idea is to have a range of commitments that conference participants of different types can relate to and that acknowledge the role of both the individual participant as well as groups and organizations in playing an important role in implementation. | | |
| **Dorothy Strachan, Facilitator (______ minutes)** | | |
| ***Tasks*** | *Total Time* | *Comments* |
| - Introduces the Session Chair Panel |  |  |
| Take home points: | | |
| **Graham Creasey, Praxis 2016 Chair, Product Development (______ minutes)** | | |
| ***Tasks*** | *Total Time* | *Comments* |
| - Made 2-3 points related to insights and recommendation for action |  |  |
| - Closes discussion with “Here is what I am prepared to do to support addressing the gaps discussed in relation to Valleys 1 and 2 throughout the conference.” |  |  |
| - Engages in ‘fireside’ discussion other speakers at end. |  |  |
| Take home points: | | |
| **Naomi Kleitman, Chair Session 2 – Pre-clinical and Clinical Trials of Regeneration and Repair in SCI (______ minutes)** | | |
| ***Tasks*** | *Total Time* | *Comments* |
| - Made 2-3 points related to insights and recommendation for action |  |  |
| - Closes discussion with “Here is what I am prepared to do to support addressing the gaps discussed in relation to Valleys 1 and 2 throughout the conference.” |  |  |
| - Engages in ‘fireside’ discussion other speakers at end. |  |  |
| Take home points: | | |
| **Catherine Truchon, Chair Session 3 – Implementing Research Knowledge in the Development of Leading Practice (______ minutes)** | | |
| ***Tasks*** | *Total Time* | *Comments* |
| - Made 2-3 points related to insights and recommendation for action |  |  |
| - Closes discussion with “Here is what I am prepared to do to support addressing the gaps discussed in relation to Valleys 1 and 2 throughout the conference.” |  |  |
| - Engages in ‘fireside’ discussion other speakers at end. |  |  |
| Take home points: | | |
| **Lisa MacKerracher, Chair Session 4 – Financial Viability – Uncertain Markets: Investors, Industry, Intellectual Property and Insurance: – Product Development and Reimbursement (______ minutes)** | | |
| ***Tasks*** | *Total Time* | *Comments* |
| - Made 2-3 points related to insights and recommendation for action |  |  |
| - Closes discussion with “Here is what I am prepared to do to support addressing the gaps discussed in relation to Valleys 1 and 2 throughout the conference.” |  |  |
| - Engages in ‘fireside’ discussion other speakers at end. |  |  |
| Take home points: | | |
| **Closing Remarks: Graham Creasey, Praxis 2016 Chair, Product Development (______ minutes)** | | |
| ***Tasks*** | *Total Time* | *Comments* |
| - Thanks conference participants |  |  |
| - Discusses briefing note |  |  |
| - Discusses press releases and social media |  |  |
| - Discussion conference report writers and session output leads next steps |  |  |
| - Acknowledges speakers and their engagement prior to the conference. |  |  |
| Take home points: | | |
| **Closing Remarks: Bill Barrable, RHI (______ minutes)** | | |
| ***Tasks*** | *Total Time* | *Comments* |
| - Thank you to conference teams |  |  |
| - Lists commitments and who will be accountable |  |  |
| - Highlights evaluation |  |  |
| Take home points: | | |
