# Supplementary material for: RE-AIMing conferences: evaluating the adoption, implementation and maintenance of the Rick Hansen Institute’s Praxis 2016
Source: Health Res Policy Syst. 2019 Apr 11;17:39. doi: 10.1186/s12961-019-0434-1 (PMC6458740; doi:10.1186/s12961-019-0434-1)
Supplement: Supplementary file 4 — Implementation goals. (DOCX 19 kb) [file 12961_2019_434_MOESM4_ESM.docx]

Table 1. Completion of Praxis Session Goals

| **Opening Session** | **Completion of goal based on > 50% of evaluators marking the goal as complete** |
| --- | --- |
| *Broad Goals* |  |
| Goal 1: participants welcomed to 1^st^ Praxis | ✓ |
| Goal 2: sharpen focus on conference objectives | ✓ |
| Goal 3: invited to poster presentations | ✓ |
| Goal 4: encouraged introductions at tables | X |
| *Speaker Goals (8 Speakers)* |  |
| Goal 1: discuss challenges/barriers have/are facing in work/personal experiences | ✓ |
| Goal 2: discusses solutions to challenge barriers | ✓ |
| Goal 3: discusses how their learnings can be leveraged into discussion of concrete actions | ✓ |
| **Session 1** |  |
| *Broad Goals* |  |
| Goal 1: sets expectations for flow of Praxis | ✓ |
| Goal 2: discusses handout | ✓ |
| Goal 3: discusses guidelines for working together | ✓ |
| Goal 4: introduce session and panel | ✓ |
| *Speaker Goals (4 Speakers)* |  |
| Goal 1: provides 2-3 challenges/barriers to implementation | ✓ |
| Goal 2: provides key learning for next time | ✓ |
| Goal 3: does not use powerpoint | X |
| **Session 2** |  |
| *Broad Goals* |  |
| Goal 1: presents case study based on experience | ✓ |
| Goal 2: named 2-3 barriers in handout | ✓ |
| Goal 3: mentions how to overcome barriers in hindsight | X |
| Goal 4: provides what needs to be done in future | X |
| Goal 5: does not use powerpoint | X |
| *Speaker Goals (3 Speakers)* |  |
| Goal 1: presents case study based on experience | ✓ |
| Goal 2: named 2-3 barriers in handout | X |
| Goal 3: mentions how to overcome barriers in hindsight | ✓ |
| Goal 4: provides what needs to be done in future | ✓ |
| Goal 5: does not use powerpoint | X |
| **Session 3** |  |
| *Facilitator Discussion Tasks* |  |
| Task 1: introduce session and panel | X |
| Task 2: discusses group results from yesterday | ✓ |
| Task 3: overview of key actions & solutions | X |
| Task 4: discusses implications for today | ✓ |
| Task 5: same as task 1 | ✓ |
| *Speaker Goals (3 Speakers)* |  |
| Goal 1: provides what works and what does not work in regards to implementation | X |
| Goal 2: provides what needs to happen to facilitate implementation | ✓ |
| Goal 3: does not use PowerPoint | X |
| **Session 4** |  |
| *Facilitator Discussion Tasks* |  |
| Task 1: introduces session | ✓ |
| Task 2: introduces panel and topic | ✓ |
| Task 3: discusses implications for today | X |
| *Table Introduction Tasks* |  |
| Task 1: tables engaged in introductions | X |
| *Speaker Goals (3 Speakers)* |  |
| Goal 1: provides 2-3 challenges | ✓ |
| Goal 2: provides primary solution to address challenges | ✓ |
| Goal 3: outlines what key 1 or 2 driving forces would support implementation of solution | X |
| Goal 4: outline 1 or 2 key restraining forces needing to be addressed and how to address | X |
| Goal 5: does not use powerpoint | ✓ |
| **Closing Session** |  |
| *Facilitator Discussion Tasks* |  |
| Task 1: introduces the session chair panel | ✓ |
| *Speaker Goals (4 Speakers)* |  |
| Goal 1: made 2-3 points related to insights and recommendation for action | ✓ |
| Goal 2: closes discussion with certain quote | ✓ |
| Goal 3: engages in ‘fireside’ discussion other speakers at end | X |
| *Closing Remarks 1* |  |
| Task 1: thanks conference participants | ✓ |
| Task 2: discusses briefing notes | ✓ |
| Task 3: discusses press releases & social media | X |
| Task 4: discusses conference report writers & session output leads next steps | ✓ |
| Task 5: acknowledge speakers & their engagement prior to conference | ✓ |
| *Closing Remarks 2* |  |
| Task 1: thank you to conference teams | ✓ |
| Task 2: lists commitments & who will be accountable | ✓ |
| Task 3: highlights evaluation | ✓ |

Table 2. Completion of Praxis Panel Goals

| **Session 1 Panel Goals** | **Completion of goal based on > 50% of evaluators marking the goal as complete** |
| --- | --- |
| Goal 1: panelists discuss key points/remarks of other speakers | X |
| Goal 2: panelists discuss areas of agreement | X |
| Goal 3: panelists discuss areas of disagreement | X |
| Goal 4: panelists discuss promising solutions | ✓ |
| **Session 2 Panel Goals** |  |
| Goal 1: panelists discuss what stands out with respect to future change | X |
| Goal 2: panelists discuss key improvements | X |
| **Session 3 Panel Goals** |  |
| Goal 1: ask Anthony & Cathy about what they heard from Mark that can be applied to SCI | ✓ |
| Goal 2: discuss what promising practices are not in general use | ✓ |
| Goal 3: plenary Q&A emerges from panel discussion | ✓ |
| **Session 4 Panel Goals** |  |
| Goal 1: discusses questions from groups | ✓ |
| Goal 2: discuss aspects to round out speakers proposals | ✓ |
| Goal 3: session chair adds to discussion | ✓ |
